# Prevalence and predictors of urinary tract infection and severe malaria among febrile children attending Makongoro health centre in Mwanza city, North-Western Tanzania

**DOI:** 10.1186/0778-7367-70-4

**Published:** 2012-03-16

**Authors:** Bahati P Msaki, Stephen E Mshana, Adolfina Hokororo, Humphrey D Mazigo, Domenica Morona

**Affiliations:** 1 Department of Pediatrics, Bugando Medical Centre/Catholic University of Health and Allied Sciences, P.O. Box 1464, Mwanza, Tanzania; 2 Department of Pediatrics, Sekou Toure Regional Hospital, P.O. Box 132, Mwanza, Tanzania; 3 Department of Medical Microbiology/Immunology, Weill School of Medicine, Catholic University of Health and Allied Sciences, P.O. Box 1464, Mwanza, Tanzania; 4 Department of Medical Parasitology and Entomology, School of Medicine, Catholic University of Health and Allied Sciences, P.O. Box 1464, Mwanza, Tanzania

**Keywords:** Fever, Malaria, Urinary tract infection, Bacteremia, Under-fives, Tanzania

## Abstract

**Background:**

In malaria endemic areas, fever has been used as an entry point for presumptive treatment of malaria. At present, the decrease in malaria transmission in Africa implies an increase in febrile illnesses related to other causes among underfives. Moreover, it is estimated that more than half of the children presenting with fever to public clinics in Africa do not have a malaria infection. Thus, for a better management of all febrile illnesses among under-fives, it becomes relevant to understand the underlying aetiology of the illness. The present study was conducted to determine the relative prevalence and predictors of *P. falciparum *malaria, urinary tract infections and bacteremia among under-fives presenting with a febrile illness at the Makongoro Primary Health Centre, North-Western Tanzania.

**Methods:**

From February to June 2011, a cross-sectional analytical survey was conducted among febrile children less than five years of age. Demographic and clinical data were collected using a standardized pre-tested questionnaire. Blood and urine culture was done, followed by the identification of isolates using in-house biochemical methods. Susceptibility patterns to commonly used antibiotics were investigated using the disc diffusion method. Giemsa stained thin and thick blood smears were examined for any malaria parasites stages.

**Results:**

A total of 231 febrile under-fives were enrolled in the study. Of all the children, 20.3% (47/231, 95%CI, 15.10-25.48), 9.5% (22/231, 95%CI, 5.72-13.28) and 7.4% (17/231, 95%CI, 4.00-10.8) had urinary tract infections, *P. falciparum *malaria and bacteremia respectively. In general, 11.5% (10/87, 95%CI, 8.10-14.90) of the children had two infections and only one child had all three infections. Predictors of urinary tract infections (UTI) were dysuria (OR = 12.51, 95% CI, 4.28-36.57, *P *< 0.001) and body temperature (40-41 C) (OR = 12.54, 95% CI, 4.28-36.73, *P *< 0.001). Predictors of *P. falciparum *severe malaria were pallor (OR = 4.66 95%CI, 1.21-17.8, *P *= 0.025) and convulsion (OR = 102, 95% CI, 10-996, *P *= 0.001). *Escherichia coli *were the common gram negative isolates from urine (72.3%, 95% CI, 66.50-78.10) and blood (40%, 95%CI, and 33.70-46.30). *Escherichia coli *from urine were 100% resistant to ampicillin, 97% resistant to co-trimoxazole, 85% resistant to augmentin and 32.4% resistant to gentamicin; and they were 100%, 91.2% and 73.5% sensitive to meropenem, ciprofloxacin and ceftriaxone respectively.

**Conclusion:**

Urinary tract infection caused by multi drug resistant *Escherichia coli *was the common cause of febrile illness in our setting. Improvement of malaria diagnosis and its differential diagnosis from other causes of febrile illnesses may provide effective management of febrile illnesses among children in Tanzania

## Background

The etiology of fever in areas where it can be due to multiple causes brings serious challenges in management, especially in areas where presumptive treatment is the norm [1]. For many years, fever has been used as the entry point for presumptive treatment of malaria in African children, with majority of these children given antimalarial drugs [2]. However, there is evidence of change in the malaria epidemiology in Africa characterized by decreased transmission, morbidity and mortality [3-5]. The epidemiological changes observed are attributed to the increase in the use of malaria rapid tests for diagnosis, the introduction of artemesinin-based combination therapy (ACT), the use of insecticides-treated nets (ITNs) and insecticides residuals spraying (IRS) [3-5]. A decline of *P. falciparum *prevalence rates in children aged 2-10 years from 37% before the year 2000 to 17% after 2000 has been documented [6]. In addition, surveys conducted since 2004 have shown a decline in under-five children mortality estimates over the past five years by an average of 23% [7]. A reduction of malaria transmission implies that there is also a decline in proportion of fever attributable to this infection among children in Africa, although this relationship is not linear [8]. Alternatively, the decline in malarial attributable fever implies an increase in febrile illnesses related to causes other than malaria in malaria endemic countries [1].

It is estimated that more than half of the children presenting with fever to public clinics in Africa do not have a malaria infection [9]. Thus, presumptive treatment of fever is not justifiable [5], as the causes of fever cannot be attributed exclusively to malaria but also to other infections due to bacteria and viruses [1]. A more accurate case management, especially among under-fives living in areas with low transmission of malaria, requires laboratory evidence on the likely causes of fever [1,10,11]. This will support clinicians in their choice of treatment options and will enable them to report more accurately on the aetiology of febrile illnesses in children. In addition, diagnosis based on laboratory evidence will reduce the unnecessary or inappropriate use of antimalarials or antibiotics and allow proper treatment for each specific cause of fever, in turn reducing the morbidity and mortality of children [5].

At the Makongoro Health Center in Mwanza, Tanzania, presumptive treatment of all fever with antimalarial and antibiotics is a common practice and is supported by the guidelines of the Integrated Management of Childhood Illness (IMCI) programme. IMCI was introduced in 1996 in primary health care facilities [12,13] where laboratory services for the diagnosis of other causes of fever such as bacterial and viral infections were unavailable. As a result, the aetiology of fever among under-fives attending health facilities often remains inaccurate or unknown. Infections with multiple diseases with similar clinical signs and symptoms bring serious challenge to clinicians, especially when there is no laboratory evidence to support the treatment decision. In other settings, clinician's behaviors have been found to influence treatment decisions [14]. The present study was conducted to determine the prevalence and predictors of *P. falciparum *malaria, urinary tract infections (UTI) and bacteremia among under-fives presenting with febrile illnesses at the Makongoro Health Centre, North-Western Tanzania, thus establishing the differential of fever attributable to either condition. In addition, the study investigated the antimicrobial susceptibility pattern of the isolates from blood and urine samples.

## Methods

### Study area

The study was conducted at the Makongoro Health Center, a primary health care facility located in Nyamagana District within Mwanza City, Tanzania. Approximately 17,431 people seek health services at this health facility per year. Over 50% of the patient population seeking services is under-fives.

Mwanza City lies on the southern shores of Lake Victoria at an altitude of 1140 m above sea level. The basin is characterized by a tropical and subtropical climate with temperatures varying from 18°C to 20°C during the rainy season and 26°C to 30°C during the dry season. Annual rainfall ranges from 700-1000 mm with long rain seasons occurring from February to May. The Lake Victoria basin is located in the holoendemic area for malaria transmission with intense transmission observed during the long rain season (February-May) and at the end of the long rain season (May-June).

### Study design

A cross-sectional analytical survey was conducted from February to June 2011. During this period, blood and urine cultures were obtained from all under-fives who fulfilled the inclusion criteria. In addition, thin and thick blood smears were obtained for malaria diagnosis.

### Study population, inclusion and exclusion criteria

All children aged two months to five years of age presenting with a febrile illness at the health facility were recruited for the study. The inclusion criteria were age between two months to five years, fever (defined as axillary temperature ≥ 37.5°C) and parents/guardians/caregivers willing to give written informed consent for their children to participate in the study. On the other hand, children were excluded if they presented with a history of use of antimalarials or antibiotics for the past seven days.

### Sample size determination

The required sample size for the study was calculated using a formula for a single population proportion. Kish and Leslie formula [15] was used to estimate the sample size using the prevalence of urinary tract infection and malaria of 13%. [16]. Taking critical value at 95% confidence level (Zα/2 =1.96) and degree of precision of 5%. A total of 231 underfives were recruited in the study.

### Laboratory methods

#### Blood culture

Blood culture was performed as previously described in the literature [17]. Briefly, 2 mL to 3 mL of venous blood was taken and added into 10 mL of Brain Heart Infusion Broth (BHI) (Oxoid, UK) and transported to the microbiology laboratory for incubation at 37 C and subsequent processing. All samples were sub-cultured blindly in 5% sheep blood agar, chocolate agar and Mackonkey agar after 24 h, 48 h, 96 h and 7 days [18]. Isolates were identified according to standard operative procedures [17-19].

#### Urine collection and analysis

Urethral catheterization methods were used in infants and pre-toilet trained children to collect urine samples [16,20]. For the other group of children (> 2 years), a clean catch method of the mid-stream urine was used to obtain the samples [20]. Urine samples were inoculated on cysteine lactose electrolyte deficient agar (CLED), Mackonkey and blood agar plates (Thermofisher UK, England). Urinary tract infections were diagnosed as described previously [18,21].

#### Antimicrobial susceptibility testing

Antimicrobial susceptibility pattern was determined by disk diffusion method according to CLSI [22]. The following antimicrobial agents were tested for gram positive bacteria, penicillin G (10U), ampicillin (10 μg), clindamycin (2 μg), erythromycin (15 μg), vancomycin (30 μg), ciprofloxacin (5 μg), oxacillin (5 μg) and cefoxitin (30 μg). For gram negatives bacteria discs used were ampicillin (10 μg), amoxycillin/clavunate (20/10 μg), ciprofloxacin (5 μg), tetracycline (30 μg), gentamicin (10 μg), co-trimoxazole and ceftriaxone (30 μg). Other reserve discs included ceftazidime (30 μg), cefepime (30 μg) and meropenem (10 μg) (Oxoid, UK).

### Laboratory diagnosis for malaria parasites

A drop of venous blood obtained for culture was used for preparation of thick and thin blood films for malaria parasites examination. The prepared blood slides were fixed in absolute ethanol (only the thick smear) and stained in 10% Giemsa (Sigma Aldrich, Nairobi). The slides were examined under a light microscope using objective × 100 under oil-immersion for any malaria parasite stages. The thick film served to confirm the presence or absence of the *Plasmodium *parasite, whereas the thin film was used to identify the *Plasmodium *species. A slide was considered negative for malaria parasite if no parasites were seen in at least 100 oil-immersion fields on the thick film [23]. Parasite density was determined per 200 leukocytes and then expressed using the formula described elsewhere [23].

For quality control purposes, all blood smears that were positive for malaria and a 10% sample of slides that were considered negative were subsequently re-examined by a second independent microscopist. In case of any disagreement between the first and second microscopist, the issue was resolved by a third microscopist. Malaria parasitaemia was categorized as follows: 1-500/μL, 501-1000/μL and > 1,000/μL [24].

### Patients management

All patients enrolled in the study were managed according to the Tanzanian guidelines for malaria and bacterial infection. Antibiotic therapy was reassessed according to culture results and sensitivity patterns.

### Ethical approval

Ethical approval was obtained from Weill-Bugando University College of Health Sciences/Bugando Medical Centre, Institution Review Board. Further ethical clearance was obtained from the regional health and administrative authorities of Mwanza region. For participation of the underfives, parents/guardians/caregivers were informed about the study objectives and gave informed written consent prior to inclusion into the study.

### Data analysis

Data entry was done using Microsoft Access (Microsoft Corp., Redmond, WA, USA) and analyzed using STATA version 11 (Stata Corp., College, TX, USA). Statistical test between dependent and independent variables was done by using Chi-squared test (χ^2^). Univariate and multivariate model were developed to determine the independent predictors (signs, symptoms and demographic factors) of *P. falciparum *malaria, urinary tract infections and bacteremia. The model reconsidered the significance of each risk factor by stepwise backward logistic regression at a *P*-value of < 0.2 for inclusion in the final multivariate model. Odds ratios with 95% Confidence Intervals were used to measure the strength of association at statistical significance level of *P *< 0.05.

## Results

### Demographic characteristics of the study population

**Figure 1:**
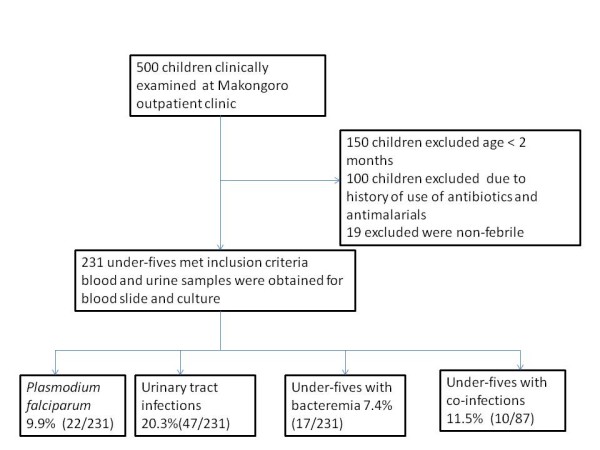
**Flow chart summarizing the number of children seen at Makongoro clinic during the study period and infections prevalences among 231 under-fives in Mwanza, Tanzania 2011**.

A total of 231 underfives with febrile illness (axillary temperature ≥ 37.5 C) were recruited (Figure 1). Among the recruited children, the median age was 15 months (inter quartile range (25th and 75th quartiles of 9 and 34 months respectively). Female formed the largest study population (54.5%, 126/231).

### Prevalence of infections

#### Prevalence and intensity of malaria

Parasitological diagnosis of malaria parasites revealed that out of the 231 underfives, only 9.5% (22/231, 95% CI, 5.72 - 13.28) had a blood slide for malaria parasite positive. All the malaria infections were due to *P. falciparum*. Of the children with a blood slide positive for malaria parasites, 77.3% (17/22, 95% CI, 71.9 - 82.70) had severe malaria as per WHO definition [25]. The prevalence of malaria was 6.7% (95% CI, 0-17.49) and 11.9% (95% CI, 5.00 - 18.80) in males and female respectively. There was no significant difference between sex (χ^2 ^= 1.8, *P *= 0.2). Among the *P. falciparum *positive children, 4.5% (1/22) had 1-500 parasites/μl, 4.5% (1/22) had between 501-1,000 parasites/μl and 90.9% (20/22) had ≥ 1000 parasites/μl.

#### Frequency of bacteremia and bacteriological results

The proportion of blood cultures that yielded a clinically significant positive result was 7.4% (17/231, 95%CI, 4.00- 10.78) among febrile under-five children. The distribution of bacteremia by sex was 9.5% and 5.6% in male and female children respectively. However, the observed difference in the prevalence of bacteremia between sex was not statistically significant (χ^2 ^= 1.3, *P *= 0.3).

*Enterobacteriaceae *were isolated in 58.8% (10/17, 95%CI, 35.5 - 82.20) of the positive blood cultures. Among them, the majority were *Escherichia coli *(4/10, 40%) and the other isolates were *Klebsiella pneumoniae *(10%), *Salmonella *species (2/10, 20%) and Gram Negative Rods (3/10, 30%). Of all the positive blood cultures, gram positive were isolated in 41.2%, with *Staphylococcus aureus *(4/7, 57.1%) and coagulase-negative *Staphylococci *(3/7, 42.9%) being the only isolates.

#### Frequency of urinary tract infection and bacteriological results

The overall prevalence of urinary tract infection was 20.3% (47/231) with female under-fives having the highest prevalence (males *versus *female: 12.4% *versus *26.9%, *P *< 0.01). *Escherichia coli *were isolated from the majority of urine samples 34 (34/47, 72.3%). Other isolates were *K. pneumoniae *(21.28%), *Proteus *species (4.26%) and *Pseudomonas spp *(2.13%).

#### Multiple infections of P. falciparum malaria, bacteremia and urinary tract infections

In general, 37.6% (87/231, 95%CI, 31.4-43.85) of all the children had *P. falciparum *malaria, urinary tract infection and bacteremia. Of all the children, 11.5% (10/87) had two infections with the majority having co-infections of *P. falciparum *malaria and urinary tract infection (60%, 6/10). Other dual infections observed were between urinary tract infections and bacteremia (3/10, 30%) and *P. falciparum *malaria and bacteremia (10%, 1/10).

#### Antimicrobial susceptibility pattern

A high prevalence of resistance (97%-100%) to ampicillin and co-trimoxazole especially to *Escherichia coli *isolated from blood and urine samples was observed. In addition, *Escherichia coli *from urine were 32.4% resistant to gentamicin. However, the *Escherichia coli *and *Klebsiella spp *isolates from urine were between 74%-100% susceptible to meropenem, ceftriaxone, ciprofloxacin and ceftazidime, while those from blood (*E. coli *isolates) were 100% sensitive to gentamicin, ciprofloxacin, ceftriaxone and meropenem.

For gram positive isolates from blood samples, *Staphylococcus aureus *isolates were 75% resistant to clindamycin and erythromycin.

#### Predictors of urinary tract infections, bacteremia and severe P. falciparum malaria

In univariate analysis, *P. falciparum *malaria (parasites ≥ 1000 parasites/μl) were strongly associated with young age, body temperature (40 C - 41 C), palmar pallor and convulsion (Table 1). However, in multivariate analysis, only palmar pallor (OR = 4.66 95%CI, 1.21 - 17.8, *P *= 0.025) and convulsion (OR = 102, 95%CI, 10 - 996, *P *= 0.001) remained significantly associated with severe malaria.

**Table 1:** Univariate and multivariate analysis for predictors of *P.falciparum *malaria for 231 underfives children attending Makongoro health centre, Mwanza city, Tanzania 2011

Variable	Malaria		Univariate			Multivariate		
	**Positive**	**Negative**	**OR**	***P*-value**	**95% CI**	**OR**	***P-*value**	**95%CI**

**Age in years**								

1-5 years	18(13.4)%	116(86.6%)	1	0.024		1		

2 m-1 year	4(4.1%)	93(95.9%)	3.61		1.18-11.03	1.44	0.42	0.42-5.1

**Sex**								

Male	7(6.7%0	98(93.3%)	1			1		

female	15(11.9)%	111(81.1)%	1.90	0.18	0.74-4.83	2.37	0.176	0.67-8.4

**Vomiting**								

No	13(8.0%)	149(92.0)	1			1		

yes	9(13.0%)	60(87.0%)	1.72	0.24	0.7-4.23	1.22	0.758	0.331-4.57

**Axillary temperature**								

37.5-39	0(0.0%)	158(100%)	N/A	< 0.001	N/A	N/A	N/A	N/A

40-41	22(30.10)	51(69.9%)						

**Palmar Pallor**								

No	12(5.9%)	191(94.1%)	1	< 0.001		1		

Yes	10(35.7%)	18(64.3%)	8.84		3.36-23.29	4.66	0.025	1.21-17.8

**Convulsion**								

No	12(5.40%)	208(94.5%)	1	< 0.001		1		

Yes	10(90.9%)	1(9.1%)	173		20-1469	102	0.001	10-996

*OR *= Odd ratio, *CI *= Confidence Interval

For urinary tract infections, in univariate analysis, dysuria, body temperature (40 C - 41 C), female sex and abdominal pain were found to be associated with urinary tract infections. On multivariate analysis only; dysuria (OR = 12.51, 95%CI, 4.28-36.57, *P *< 0.001), body temperature (40 C-41 C) (OR = 12.54, 95%CI, 4.28-36.73, *P *< 0.001) and female sex (OR = 3.40, 95%CI, 1.23-34, *P *= 0.01) remained independently associated with urinary tract infections (Table 2). No factors were found to predict bacteremia.

**Table 2:** Predictors of urinary tract infections among 231 febrile children underfives attending Makongoro health centre, Mwanza city, Tanzania 2011

Variable	UTI		Univariate			Multivariate		
	Positive	Negative	OR	*P*- value	95%CI	OR	*P*-value	95%CI
**Age in years**								
1-5 years	27(20.1%)	107(79.9%)	1			1		
2 m-1 year	20(20.6%)	77(29.4%)	1.03	0.93	0.54-1.97	1.29	0.62	0.47-3.54
**Sex**								
Male	13(12.4%)	92(87.6%)	1			1		
Female	34(27.0%)	92(73.0%)	2.62	0.07	1.30-5.27	3.40		1.23-3.34
**Dysuria**								
No	7(4.4%)	153(95.6%)	1			1		
Yes	40(56.3%)	31(43.7%)	28.2	< 0.001	11.57-68.74	12.51	< 0.001	4.28-36.57
**Axillary temperature**								
37.5-39	7(4.4%)	151(95.6%)	1			1		
40-41	40(54.8%)	33(45.2%)	26.15	< 0.001	10.77-63.48	12.54	< 0.001	4.28-36.73
**Abdominal pain**								
No	9(6.7%)	126(93.3%)	1			1		
Yes	38(39.6%)	58(60.4%)	9.17	< 0.001	4.16-20.21	1.61	0.39	0.54-4.82

*OR *= Odd ratio, *CI *= Confidence Interval

## Discussion

The present study documents the frequent occurrence of urinary tract infections, *P. falciparum *malaria and bacteremia among febrile under-fives attending a primary health care facility in North-Western Tanzania. The study has shown that the three infections are prevalent among febrile underfives and a number of factors remained independently associated with these infections.

In this study, 9.5% of all the study population had malaria positive slides, of which all were infections with *P. falciparum*. This species is known to cause almost 95% of all malaria cases in the country [26]. The prevalence of *P. falciparum *malaria observed in the present study was lower than the 12% recently reported among under-fives in Western Tanzania [26]. In Gabon, about 40% of the children presenting at a hospital with fever or history of fever had a *P. falciparum*-positive blood film [27]. The variation in malaria transmission, the use of malaria prevention tools and the season in which the study has been conducted could in part account for the observed difference in *P. falciparum *prevalence.

The prevalence of urinary tract infections of 20.3% observed among the under-fives of the present study was considerably higher than 3.3% and 9% reported in developed countries [28] and lower than 29.3% and 39.7% recently reported in East Africa [14,19]. The disparity between the present findings and those of other studies can to some extent be explained by the co-existence of different risk factors between the population under study, such as home crowding, low hygienic standards and a high prevalence of other diseases and conditions such as HIV infection and malnutrition, as observed in previous research [16]. In some studies [16,21]; *Escherichia coli *has been found to be the commonest organism isolated from urine samples and the isolates have been shown to account for up to 75% of urinary tract infections in all pediatric age groups [21], followed by *Klebsiella spp, Proteus spp *and *Pseudomonas *species [16,21,28].

The bacteremia prevalence observed here was comparable to other similar studies in Africa whereby the prevalence ranged between 7.1 - 12.2% [14,29,30]. However, the prevalence of bacteremia reported from other African countries, for instance rural Central Africa, was 24.4% [25]. In industrialized countries and in South Africa, bacteremia appears to be less frequent among children where only 3 to 8% of the febrile children had positive blood cultures [31,32]. A possible explanation for the observed disparity in the prevalence of bacteremia could be the variations in risk factors associated with the disease [16].

In contrast to studies conducted in other developing countries [33], gram-positive bacteria were less commonly isolated than gram negative bacteria. This finding was similar to what was observed in Mozambique and Kenya [16,34]. The higher frequency of bacteremia found in the present study probably illustrates the added burden of infections caused by gram negative bacteremia in the study area. *Haemophilus influenzae *and *Streptococcus pneumoniae *were not isolated in this study which could partly be explained by the technique used in our study. In the case of *Haemophilus influenzae*, the use of HIB vaccine among under-fives and seasonal variation could possibly explain the negative result.

Co-occurrences of multiple diseases in an African setting are norm rather than exception [16]. In the present study, 11.5% of the children had dual infections with majority having *P. falciparum *malaria and urinary tract infections, and least had urinary tract infections and bacteremia. These findings were similar to previous findings in African settings [16,34,35].

The commonest isolate from urine and blood was *Escherichia coli*; this isolate was found to be resistant to commonly used antibiotics like ampicillin, co-trimoxazole, amoxycillin/clavulanate and gentamicin. In addition, more than 20% of these strains were resistant to third generation cephalosporins. A recent similar study within the region has reported a resistance rate of up to 98.4% and 95.3% of *Escherichia coli *isolated from urine samples among febrile children [21]. The study also reported a higher rate of antibiotics resistance in *Klebsiella pneumoniae *as compared to *E. coli *[21]* *. Fortunately, all the organisms causing urinary tract infections and bacteremia observed here remained sensitive to meropenem and about 95% of them are still sensitive to ciprofloxacin. As a matter of fact, meropenem is an expensive injectable beta-lactam antibiotic and is not available in many drug stores in Tanzania. It is therefore less misused. As for ciprofloxacin, it is not commonly used in children below 12 years. As this study recruited patients were from primary health care facility, the high resistance rate of *Escherichia coli *to ampicillin, co-trimoxazole and augmentin is possibly indicating a problem with these particular isolates within the community. This could be explained by an inappropriate use of these antibiotics in the community, i.e. wrong prescription or poor adherence. Furthermore, the 32.4% and 26.5% resistance rates of *Escherichia coli *to gentamicin and ceftriaxone is not low for community isolates; this observation calls for a continuous local epidemiologic surveillance to monitor the resistance patterns of these antibiotics within the community.

Several factors have been independently associated with severe *P. falciparum *malaria in under-fives [36,37]. The identified predictors are fever, duration of fever, intermittent fever, vomiting, convulsion and anaemia [36,38]. In our study, palmar pallor and convulsion were associated with severe malaria. It appears that specific clinical signs or symptoms which can accurately predict malaria infection in children are difficult to identify. In Gambia, the use of fever or history of fever resulted in over-diagnosis of malaria [37]. Clearly, for the benefit of the patients and the improvement of the management of febrile conditions in under-fives, malaria management should rely uniquely on precise differential diagnoses, especially at a time when malaria transmission appears to decline in Africa [26].

A number of previous studies have reported various demographic, clinical signs and symptoms associated with urinary tract infections in children [21,28,39]. Factors such as age (< 2 years), prolonged fever (≥ 5 days), sex and heamaturia have been reported as independent risk factors for urinary tract infections in children [21,28,39]. In the present study, high fever (body temperature 40-41 C), dysuria and female sex remained as significant predictors for urinary tract infections in children. Our observations were similar to reports of other studies [21,39].

Our study is however subject to some limitations. Using a cross-sectional survey design makes it difficult to clearly show the temporal relationship between urinary tract infections, malaria and bacteremia with other variables. In addition, HIV status was not investigated in the present study.

## Conclusion

In conclusion, urinary tract infections caused by multi drug resistant *Escherichia coli *was identified as the most common cause of fever, which co-existed with malaria and bacteremia. Several independent factors were found to be associated with UTI and malaria. The isolates identified from urine were highly resistant to commonly used antibiotics in our setting. The growing resistance to commonly used antibiotics calls for the necessity of a continuous epidemiologic surveillance in primary health care facilities in Tanzania. In addition, improvement of the diagnosis of UTI and other causes of febrile illnesses will provide more effective measures in the management of febrile illnesses among under-fives in Tanzania

## Competing interests

The authors declare that they have no competing interests.

## Authors' contributions

BM participated in collecting specimens, collecting clinical data and follow up of the patients, SEM participated in design of the work, performing microbiological procedures, data analysis, interpretation of data and writing of the manuscript, AH participated in recruitment, patients management and manuscript writing, HM participate in the design of the work, data analysis and manuscript writing, DM participated in interpretation of the results and manuscript writing. All authors have read and approved the final manuscript.
